# Genome sequence of *Eubacterium callanderi* AM6, isolated from a Parkinson’s disease patient

**DOI:** 10.1128/mra.00039-25

**Published:** 2025-03-13

**Authors:** Madhav Kumar, Lizbeth Sayavedra, Dave J. Baker, Yogesh S. Shouche, Arjan Narbad

**Affiliations:** 1 Quadram Institute Bioscience, Norwich Research Park 7308, Norwich, United Kingdom; 2 Gut Microbiome Division, SKAN Research Trust 663357 , Bengaluru, Karnataka, India; Wellesley College Department of Biological Sciences, Wellesley, Massachusetts, USA

**Keywords:** *Eubacterium callanderi*, genome, Parkinson's disease, gut microbiome

## Abstract

Here, we report the genome sequence of *Eubacterium callanderi* AM6, isolated from a fecal sample obtained from a Parkinson’s disease patient. The bacterial genome was sequenced using Illumina technology on a NextSeq 500 platform. The assembled genome of *Eubacterium callanderi* comprises 4,318,463 base pairs with a G + C content of 47.5%.

## ANNOUNCEMENT

*Eubacterium callanderi* is a non-motile, gram-positive, anaerobic gut bacterium that produces short-chain fatty acids, supporting gut health and potentially benefiting conditions like Inflammatory Bowel Disease (IBD) and metabolic syndromes (1–3). Parkinson’s disease (PD) is a progressive neurodegenerative disorder characterized by motor dysfunction, dopamine loss, and potential gut-brain axis involvement. Studying *Eubacterium callanderi* from PD patients may reveal its role in gut-brain interactions, neuroinflammation, and potential as a biomarker or therapeutic target. There is limited research on *Eubacterium callanderi* isolated from patients with PD. Here, we report the genome sequence of *Eubacterium callanderi* strain AM6, isolated from the fecal sample of a PD patient.

A fecal sample from an 86-year-old female with idiopathic PD was collected in a collection pot, with anaerobic conditions maintained using an Anaerogen sachet until processing. The sample was aliquoted and stored at −80°C in the Norwich Research Park Biorepository (52.6125° N, 1.2417° E). The sample was enriched in Postgate C broth, streaked onto Postgate C agar, and incubated at 37°C for 72 hours under anaerobic conditions. Pure colonies were obtained after at least three subcultures for 24–30 hours. Genomic DNA was extracted from 1 mL of an overnight grown culture using the Maxwell RSC Cultured Cells DNA Kit (Promega, USA) according to the manufacturer’s instructions. DNA quantity was measured using a Qubit fluorometer, and the final library was analyzed with a Qubit 3.0 and Agilent TapeStation 4200. Illumina short-read sequencing was conducted using a modified protocol with a 20-fold dilution of the DNA Prep (Flex) reagent, and sequencing was performed on a NextSeq 500 platform with 150 bp paired-end reads.

Raw paired-end reads were processed using Fastp (version 0.23.4) for quality control and filtering (4). Genome assembly was performed with SPAdes (version 3.15.5) (5), and the assembled genome’s quality was assessed using QUAST (version 5.3.0) (6). The completeness of the genome was assessed using CheckM (7), yielding a value of 99.36%. The genome of *E. callanderi* AM6 comprises 4,318,463 bp with a G + C content of 47.5%, distributed across 35 contigs, and an *N*_50_ fragment size of 703,631 bp. The genome was sequenced using 2.165 million 150 bp paired-end reads, achieving a coverage depth of 75×. Annotation using the NCBI prokaryotic genome annotation pipeline (8) identified 4,145 genes, 4,060 protein-coding genes, 51 tRNAs, 4 rRNAs, and 1 ncRNA. Average nucleotide identity (ANI) analysis revealed that *E. callanderi* AM6 shares 98.4% ANI with type strain *Eubacterium callanderi* FD (accession FRBP00000000) (9), 94.08% ANI with *Eubacterium limosum* ATCC 8486 (accession CP019962), and 89.67% ANI with *Eubacterium maltosivorans* YI (accession CP029487). No plasmids were detected using PlasmidFinder (version 2.1.6) (10). Phage analysis using PHASTEST (11) identified two intact prophage regions: a 54.27-kb region (40.31% G + C content) similar to the Clostr_phiCD146 phage and a 35.88-kb region (42.17% G + C content) similar to the Clostr_PhiS63 phage. Whole genome-based taxonomic analysis was conducted using the Type (Strain) Genome Server (https://tygs.dsmz.de) (12), which validated the AM6 isolate as *Eubacterium callanderi*. Default parameters were used except where otherwise noted.

## Data Availability

The genomes have been deposited in the NCBI under BioProject and BioSample PRJNA1200751 and SAMN45912791, respectively. The raw Illumina reads are available at the NCBI Sequence Read Archive (SRA) and can be accessed under accession number SRR31783028. The assembled Genome has been deposited at DDBJ/ENA/GenBank under the accession JBKAMR000000000.
